# Developing obesity prevention interventions among minority ethnic children in schools and places of worship: The DEAL (DiEt and Active Living) study

**DOI:** 10.1186/1471-2458-9-480

**Published:** 2009-12-21

**Authors:** Maria J Maynard, Graham Baker, Emma Rawlins, Annie Anderson, Seeromanie Harding

**Affiliations:** 1 Medical Research Council, Social and Public Health Sciences Unit, Glasgow, UK; 2 Centre for Public Health Nutrition Research, Department of Medicine, University of Dundee, Ninewells Hospital and Medical School, Dundee, UK

## Abstract

**Background:**

Childhood obesity is a major public health concern with serious implications for the sustainability of healthcare systems. Studies in the US and UK have shown that ethnicity is consistently associated with childhood obesity, with Black African origin girls in particular being more vulnerable to overweight and obesity than their White peers. Little is known, however, about what promotes or hinders engagement with prevention programmes among ethnic minority children.

**Methods/Design:**

This paper describes the background and design of an exploratory study conducted in London, UK. The aim of the study was to assess the feasibility, efficacy and cultural acceptability of child- and family-based interventions to reduce risk factors for childhood and adolescent obesity among ethnic minorities. It investigated the use of a population approach (in schools) and a targeted approach (in places of worship). We used a mixture of focus group discussions, in-depth interviews and structured questionnaires to explore what children, parents, grandparents, teachers and religious leaders think hinder and promote engagement with healthy eating and active living choices. We assessed the cultural appropriateness of validated measures of physical activity, dietary behaviour and self efficacy, and of potential elements of interventions informed by the data collected. We are also currently assessing the potential for wider community support (local councils, community networks, faith forums etc) of the intervention.

**Discussion:**

Analysis of the data is ongoing but the emergent findings suggest that while the school setting may be better for the main implementation of healthy lifestyle interventions, places of worship provide valuable opportunities for family and culturally specific support for implementation. Tackling the rise in childhood and adolescent obesity is a policy priority, as reflected in a range of government initiatives. The study will enhance such policy by developing the evidence base about culturally acceptable interventions to reduce the risk of obesity in children.

## Background

Childhood obesity is a major public health concern with serious implications for the sustainability of healthcare systems [1]. Its prevalence has doubled over the last two decades in the UK and is predicted to continue to rise [2], increasing the likelihood that Type 2 diabetes, heart disease and a range of other co-morbidities, including adverse psychosocial outcomes, will occur before or during early adulthood [3]. Studies in the US [4] and UK [5-7] have shown that ethnicity is a consistent correlate of childhood obesity, with Black African origin girls in particular being more vulnerable to overweight and obesity than their White peers. Cardiovascular risk associated with obesity also varies by ethnic origin. South Asians have a greater cardiovascular risk at lower BMI levels compared with Whites [8] and South Asian children are also more prone to central adiposity [9,10]. In the UK we do not know if the mortality risk associated with obesity is higher in ethnic minority groups but US-based studies suggest higher risk for some minority groups [11]. Type 2 diabetes and cardiovascular disease are more common among adult Black African and South Asian origin populations in the UK [12,13] and a potential worsening of obesity-related risk in their children carries implications for persisting disparities in chronic disease across generations.

Obesity has been described as a multi-factorial trait linked to both genetic and non-genetic factors [14,15]. Response to environmental 'triggers' of obesity may vary among individuals but the rise in obesity in both developed and developing countries, and the generally lower levels in the latter than the former [16], suggest that environmental rather than genetic factors are the major drivers for ethnic differences in obesity in developed countries. Lower levels of obesity and of obesity-related chronic diseases among West African populations in West Africa than in the Caribbean and the US [17] support the hypothesis that migration from developing to developed countries is associated with an increased risk of obesity. In the UK, increasing cardiovascular risk with length of residence among South Asians [18] and higher intake of fat among UK-born compared with migrant Jamaicans [19], suggest a transition from traditional dietary and physical activity (PA) habits to those that prevail in the UK. The MRC DASH study http://www.sphsu.mrc.ac.uk/study-sites/dash is one of the few studies that can be used to systematically examine adolescent and parental behavioural factors, family life and socio-economic disadvantage in relation to ethnic differences in obesity in early adolescence. In this school-based study with about 6, 000 adolescents, 80% of whom are from ethnic minorities, some of the key correlates of obesity include skipping breakfast, parental obesity and parental smoking, all of which are associated with disadvantage [20]. Ethnic minorities tend to engage more than White British adolescents in dietary practices that promote obesity. For example, compared with White British girls, Black Caribbeans, Black Africans and Pakistani girls are more likely to skip breakfast and consume carbonated drinks daily and less likely to have the recommended daily intake of fruit and vegetables. South Asian girls are least likely to take part in regular vigorous PA [20]. In addition, these data point to an important role of acculturation as obesogenic behaviours appear to be more prevalent among ethnic minority adolescents born in the UK than among those born in home countries.

Despite this increased vulnerability to obesogenic behaviours, intervention studies in the UK targeting ethnic minority children are sparse. We are aware of one prevention study currently in progress - the Birmingham healthy Eating and Active Lifestyles for Children study (BEACHeS), funded by the National Prevention Research Initiative (NPRI) - that is targeting five to six- year old South Asian children, http://www.beaches.bham.ac.uk/index.shtml. The need for effective models for obesity prevention among ethnic minority children is critical. Cultural preferences may influence engagement with different recreational activities and food choices. The nature of social support networks, essential for promoting health behaviour change, may also be different. Family-based approaches are strongly supported in the obesity treatment literature [21], as social support from family members may encourage healthy dietary adherence [22]. Compared to the Western focus on the individual, relational and family-orientated, non-Western traditions may influence the relevance and uptake of health promotion messages [23]. Ethnic-specific health intervention studies in the US have often involved both family and community organisations, promoting social support from the family and community as well as obtaining direct access to specific populations [24-26]. Religious organisations, in particular, can serve as centres of community life for ethnic minorities, and successfully promote culturally tailored health behaviour change interventions [27,28]. The DASH data suggest high weekly attendance to places of worship. For example over 84% of Nigerian and Ghanaian, 60% Other Africans, 43% Black Caribbean, 53% Indian and 69% of Pakistani/Bangladeshi 11-13 year olds in the DASH study reported weekly attendance to a place of worship compared with 9% of British Whites. Weekly attendance is generally higher among girls than boys [29]. Using a composite measure of a range of activities that children do with their parents, ethnic minority children also appear to do more activities with their parents than White British adolescents [30]. As recommended in the literature on intervention studies targeting children [14], the DASH findings suggest that in the promotion of healthy living strategies (healthy eating, active living and mental health) parental involvement should be key components of an intervention programme targeting ethnic minorities in the UK. This paper describes the study design of the UK DiEt and Active Living study.

## Methods/Design

### DEAL (DiEt and Active Living study)

The aim of DEAL is to conduct developmental research in both schools and places of worship to identify culturally acceptable child- and family-based interventions to reduce dietary and physical activity risk factors for childhood and adolescent obesity among ethnic minorities. We explore how potential interventions work and the setting most likely to support effective engagement. The study includes children aged 8-13 years which allows us to investigate how issues related to transition to early adolescence (and possibly less parental supervision) affect intervention strategies. The perspectives of parents, grandparents, teachers and religious leaders are also included. The intention is that these data will provide the basis for a pragmatic pilot Randomised Controlled Trial at a later date.

**Figure 1:**
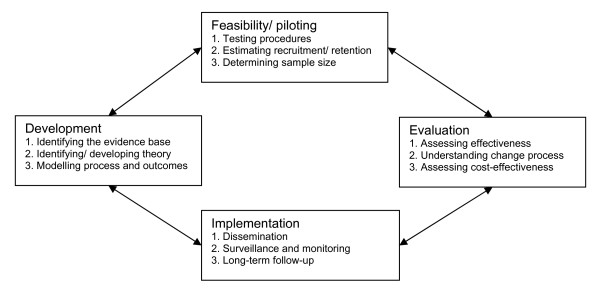
**Main stages and key functions/activities in the development and evaluation of complex interventions (adapted from Craig et al **[32]).

The design of this study draws on the principles of Green and Kreuter's [31] precede-proceed model (which emphasises that health is influenced by multiple factors and therefore methods for effecting change must be multidimensional and participatory), the MRC framework for development and evaluation of complex interventions (development, feasibility/piloting phases (Figure 1)) [32], and the 'health-promoting family' model developed by Christensen [33].

We are using a combination of qualitative and quantitative methods to explore what participants think hinders and promotes engagement with healthy eating and active living. Small scale piloting among the children of potential measures and interventions has been carried out to develop the components and intervention package. We focus on behaviours that assist children to improve energy balance - through balancing energy intake (from all forms of food and drinks) with appropriate activity levels (all forms of school and home physical activities). In particular, we are seeking to establish how best to achieve specific targets for specific behaviours (e.g. increasing overall activity levels to achieve 60 minutes of activity on most days). Data collection among the children, parents and grandparents is complete. Data analysis is ongoing and we are also currently assessing the potential for wider community support (local councils, community networks, faith forums etc) of the intervention. All DEAL information sheets, questionnaires and focus group/interview schedules may be accessed via the study website http://www.sphsu.mrc.ac.uk/studies/dash/DEAL/.

#### Identifying the evidence base

A recent systematic review by Brown and Summerbell [34] concluded that diet and physical activity interventions may help prevent children becoming overweight in the long-term. It is difficult to generalise, however, regarding which interventions are most effective due to the heterogeneity of studies and there was a lack of prevention studies targeting ethnic minority children. In collaboration with Brown and Summerbell, we extracted from their review the studies that described the interventions and the methods for measuring intermediate (e.g. self efficacy for physical activity (PA)) and outcome measures (e.g. PA and sedentary behaviours).

#### Ethical approval and consent

Approval for the study was obtained from the University of Glasgow's Medical Faculty Ethics Committee, and relevant religious organisations. All participants received a written or orally administered information sheet. Parental consent was obtained on an opt-out basis, where parents were provided with a form to be signed and returned only if they did not want their child to participate. Active consent was required from the children, parents and grandparents for their own participation. Information sheets and consent forms were translated into Urdu, Punjabi and Gujarati as well as providing them in English. Issues around consent when literacy levels are low were explored in focus groups with grandparents.

### Recruitment

We proposed to recruit six schools (three primary, three secondary) and six places of worship (two churches, two temples/mandirs and two mosques) from high minority ethnic density areas in London, ensuring involvement of individuals from six ethnic groups: Black Caribbeans, Black Africans, Indians, Pakistanis, Bangladeshis and (from schools only) White British. A short screening questionnaire was completed by each participant to ensure adequate representation by age, gender and socio-economic status within each ethnic group.

#### Schools

Three secondary schools (in the London boroughs of Brent, Lambeth and Wandsworth) with at least 15-20% of any of the ethnic groups listed above were invited to take part. Two of these schools were previously involved in the DASH study and important links existed with key contacts (head teacher, head of year) in all three schools. Following initial email and/or telephone communication with key contacts, site visits were made by the research team to inform members of staff acting as study co-coordinators within the school. All three schools agreed to take part but one later withdrew before data collection commenced, citing lack of time and conflict with the school curriculum. The three feeder primary schools of the invited secondary schools were also recruited.

#### Places of Worship

A Church of England and a Pentecostal church, one mosque, one Shree Swaminarayan temple and one Sikh Gurdwara in the boroughs of Croydon, Ealing, Hackney, Hillingdon and Lambeth, were recruited. In contrast to schools, recruiting places of worship to the study was a greater challenge. A combination of recruitment strategies including mass mailing, personal referrals, "cold calling" site visits and involvement of local authority led inter-faith groups was required. An additional mosque that had agreed to participate withdrew following initial unsuccessful recruitment of families and subsequent loss of communication with the key contact. In addition to the expected between- group heterogeneity in places of worship, there was much more variation within the ethnic groups of interest in where people worshipped, than originally anticipated (the Tamils (Hindus), Jains, Singhalese (Buddhists) and Sikhs all attend distinctly different places of worship, for example). This meant a much more differentiated approach in targeting the sample and the study was therefore extended to include a Tamil temple, a Jain community prayer group and an additional Pentecostal church.

### Sample

The aim was that at least four children from each ethnic group, two boys and two girls, would complete the diet and PA behaviour measures, yielding a sample of 44 children (24 from schools; 20 from places of worship); each focus group was ideally to comprise 6-8 individuals in the school setting and 3-4 in the places of worship. As the DEAL study is at the development and feasibility stages of complex interventions (Figure 1), the sample size was chosen pragmatically so that the ethnic groups of interest (and their concomitant places of worship) were represented. The data generated from this phase will contribute to sample size power calculations for a subsequent pilot randomized controlled trial.

Pupils in the final year of primary school (10-11 years) and first year at secondary school (11-12 years) from the ethnic groups listed above and their parents were recruited via the schools. Potential participants were identified and selected by the key contacts. The reality of attempting to work with particular ethnic groups in multi-cultural schools was that although teachers were happy to select a diverse group they did not want to select some ethnicities to the exclusion of others. Also, teachers did not always know the precise ethnicity of a child and there is a growing prevalence of mixed identity. Consequently, in addition to our target sample, we recruited an additional seven child participants from other ethnic groups e.g. Iranian, Afghani, mixed Black African/White UK (Table 1). In the places of worship participants were also identified by key contacts, but in addition some responded to general recruitment drives, were present on the study day and volunteered to take part, or entered the study via snowball recruitment. The aim was to recruit children aged 10-12 years to mirror the sample from schools. However, this age range was extended in order to achieve the required sample, as the ages of children in attendance differed by site. Black African, Black Caribbean, Indian, Pakistani and Bangladeshi families with a child aged 8-13 years were recruited. Two White UK families were also recruited, although this was not in the original aims. Grandmothers were recruited from the mosque, the Jain community prayer group and one of the Pentecostal churches. Although recruitment was generally more difficult in places of worship compared to schools, targeting by ethnic groups was inherent which in some cases resulted in over sampling, particularly for the Indians. The achieved sample of study participants by ethnicity and site is shown in Table 1; Table 2 details the focus group locations and numbers of participants. Of the 77 children who took part 58 participated in both piloting measures of behaviours and focus groups, 12 in focus groups only and 7 in measures only.

**Table 1:** Number and ethnicity of participants

Ethnicity	Schools		Places of worship		
	**Children**	**Parents**	**Children**	**Parents**	**Grandparents**
Black African	6	2	4	4	1
Black Caribbean	4	2	3	3	3
Indian	4	4	27	15	6
Pakistani	5	1	6	4	2
Bangladeshi	3	2	3	1	-
White UK	3	1	2	1	-
Mixed	4	-	-	-	-
Other	3	2	-	1	-

**Total**	**32**	**14**	**45**	**29**	**12**

**Table 2:** Focus group locations and participants

Location	Number of focus groups *(number of participants in each group*^1^)		
	**Children**	**Parents**	**Grandmothers**
Primary schools	3*(8; 7; 3)*	2 *(2; 3)*	
Secondary schools	3 *(6;4;4)*	1*(5)*	
Churches	2 *(5;3)*	2 *(5;4)*	1 (4)
Mosque	2 *(6:6)*	1 *(6)*	1 *(2)*
Sikh Gurdwara	1 *(4)*		
Hindu Temple	1 *(7)*	1 *(3)*	
Jain community centre	-		1 *(5)*
Tamil Hindu temple	1 (7)	1 (10)	

**Total**	**13*(70)***	**8 *(38)***	**3 *(11)***

^1 ^child, parent and grandparent focus groups conducted separately

### Data collection

#### Focus group discussions and interviews

Focus groups were conducted with children, parents and grandparents in each primary and secondary school, and place of worship. In contrast to schools where focus groups comprised individuals of diverse ethnicities, focus groups in places of worship were largely ethnic specific. The perceived barriers and facilitators that influence engagement with healthy food choices and sufficient PA in the different ethnic groups were explored in each group. Twenty-four focus groups were conducted, separately for children and adults as the presence of parents or other family members may have constrained the views of the children.

Topic guides were developed to cover the areas of health related knowledge and attitudes; facilitators and barriers including competing priorities, family life and parental support and access to opportunities; preferences for activities and dietary patterns. The guides were used to ensure that the key themes identified from the review of the scientific literature were covered; however, free discussion of experiences and ideas was encouraged.

The focus groups were conducted in the most appropriate space available (classroom, library) and moderated by ER, a qualitative social scientist. The sessions lasted about one hour and were tape-recorded with participants' consent. Where focus groups could not take place (e.g. in one school setting many of the parents were shift workers and arranging a group session was impossible) individual semi-structured interviews (*n *= 6) were carried out using a similar topic guide to that of the focus groups. These interviews were conducted in the participants' homes or in schools.

We found that grandmothers/mother-in-laws are key influences on dietary intake and general care of South Asian children in particular. We therefore explored how grandmothers might support family based interventions and, as stated above, examined issues related to obtaining informed consent, and the acceptability of novel methods of obtaining consent such as video recording, among older ethnic minority participants.

The focus group and interview data are transcribed and are currently being be analysed using the NVIVO software package [35]. All authors are independently coding the transcripts to verify the coding. The broad analysis categories are theoretically driven and methodological rigour maintained by an iterative process of analysis and re-analysis to identify new themes and sub-themes [36]. Particular attention is being paid to the ethnic specific contextual influences (e.g. family/other social support) on knowledge and attitudes towards healthy living and on barriers, facilitators and preferences related to healthy food choices and active living.

#### Questionnaire survey of key informants

A questionnaire survey was used to formalise consultations with teachers, PA coordinators, and religious leaders. Topics include commitment to supporting an intervention programme, skills and resources required, whether PA and food technology teachers are interested in being the interventionists, best methods of engaging both parents and children and the logistics of effective implementation in the two settings. These data will be used to develop a checklist of requirements essential for the support of an intervention programme and to develop training packages, identifying best practice to maximise parent-child involvement, and to identify barriers to implementation. Similarly, a questionnaire is being developed for wider community networks and support structures (faith groups, community voluntary organisations), local councils and primary care trusts to assess their willingness and capacity to support and/or implement an intervention and disseminate related information. A number of the target organisations have already informally indicated interest and support.

#### Piloting of interventions

Small scale piloting of potential intervention components and measures were carried out in group sessions. These sessions were followed by qualitative evaluation on a group and individual basis in order to identify the activities considered most appealing and to plan appropriate modifications. Practically oriented, interactive sessions were designed to focus on the following behaviours: 1) habit formation (e.g. breakfast consumption), 2) reduced intake of high energy-dense foods and sugary drinks and 3) increased everyday activity (and reduction of sedentary behaviour).

##### 'Taster' sessions

Sessions provided in schools and places of worship comprised a range of activities which might be plausible to include in a subsequent trial (Table 3). These sessions focused on raising awareness, improving skills and exploring goal setting as key components of obesity prevention programmes. In schools, sessions took place within a range of settings (classrooms, gym halls, playgrounds etc.) in various subject classes (food technology, physical education, Personal Social and Health Education lessons) with whole class participation. In places of worship, sessions took place in the most suitable area available (e.g. library) and groups ranged in size from 6-13 participants. The focus group and feasibility testing sample took part in the intervention sessions in their school or place of worship. Sessions were largely researcher led but we also explored engaging others (a professional dancer, local authority PA coordinators, and school staff) to support the delivery of interventions.

**Table 3:** Intervention 'taster' sessions

Diet only sessions				
**Location**	**Topic**	**Intervention session aims, content and mode of delivery**		

		**Awareness raising**	**Skill development/Learning activities**	**Delivery#**

**Primary school**	5-a day fruit and vegetables	* The five-a day message * Fruit and salad alternatives to energy dense snacks	* Making simple fruit and salad vegetables dishes; * Tasting session; * Fruit and vegetable goal setting	* Interactive knowledge sessions; * Small group work

**Secondary school**	Healthy snacks	* Incorporating dietary alternatives to energy dense drinks/snacks e.g. one smoothie per day	* Design and make fruit smoothies and savoury dips; * Tasting session	* Small group work

	Fats in the diet	The role of fats: * as part of a healthy diet; * in the energy density of foods	* Group discussion; * Worksheets; * Demonstration of types of fats * Understanding traffic light food labelling	* Powerpoint presentation; * Whole group demonstration

**POW**	Breakfast options	* Importance of eating breakfast; * The impact of skipping breakfast on food intake throughout the day	* Discussion/reflection on own habits; * Tasting session of various healthy breakfast options	* Powerpoint presentation and discussion; * Whole group activities

**PA only sessions**				

**Primary school**	Benefits of PA	* Understanding the benefits of PA; * Maintaining healthy body weight	* Circuit training- style range of activities; * Group discussion	* Local authority PA advisor-led whole group and small group activities

	Conventional sport	* Using popular activity to reinforce PA messages	* Game of football; * Group discussion	* Team game; Whole group activities

**Secondary school**	"Street dance"	* Using popular activity to reinforce PA messages	* Learning and executing a dance routine; * Group discussion	* Professional-led dance session; * Whole group activities

	PA frequency, intensity, type and time (FITT) principles	* Physical activity requirements for health; * FITT principles; * Active leisure; * Increasing PA in daily activities and reducing sedentary behaviours	* Group discussion; * Worksheets; * Goal setting (to accumulate additional 1 hr PA over individually set timeframe)	* Power-point presentation; * Small group activities

**POW**	Physical activity and energy balance	* Principles of PA; * How PA is linked to energy balance	* Group discussion; * Worksheets; * Demonstration of pulse reading	* Power-point presentation; * Small group activities; * Whole group demonstration

**Combined Diet and PA sessions**				

**Primary school**	Arterial stiffness	* Heart health; * Heart and arterial stiffness in relation to diet, PA and cardiovascular fitness	* 20 m 'shuttle run' assessment of cardiovascular fitness	* Powerpoint presentation and discussion * Small group activities

	Energy balance	* Principles of energy balance.	* Worksheets; * Interactive session with foods; * Quiz	* Powerpoint presentation; * Small group activities

# all sessions were led by researchers unless otherwise stated

The content of the intervention sessions covered the skills and knowledge required to make healthy food and activity choices such as understanding energy balance, the energy content of foods, understanding food labels, and strategies for finding time and support for PA. Sessions were practical approaches on ways to incorporate healthy eating and active living messages tailored towards the three main intervention behaviours listed above e.g. tasting novel fruit was used to emphasise the 'five a day message' and also their potential as alternatives to confectionary and other energy dense snacks. The content of sessions was also informed by emergent gaps in knowledge and the activities that respondents reported as enjoyable in the focus group discussions. Practical activities were combined with learning activities and goal setting. Sessions either had a solely diet or a PA emphasis, or a combination of the two (see Table 3). Short written individual evaluations were completed by all session participants, and focus group evaluation among the focus group/feasibility sample. All participants received interactive materials to take home such as quizzes and fact-sheets (aimed at reinforcing messages) and certificates and pens as positive feed back for taking part.

### Feasibility testing of behavioural measures

The piloting of instruments which measure dietary and PA behaviour and the potential for behavioural change in children was conducted over a number of weeks. Process evaluation of the acceptability of each measure was carried out using individual, short evaluation forms together with focus group discussions or interviews. Participants were asked to complete and evaluate a second round of measures to assess acceptability of repeat measures.

#### PA and sedentary behaviour

Based on our review of the literature, the feasibility of both subjective (self-report recall) and objective measures (accelerometry) of physical activity was explored. The accuracy of information collected by subjective instruments is influenced by the ability to accurately recall all relevant details retrospectively. Young children's activity patterns may be more variable and harder to remember than adults and cognitive and linguistic ability by age also plays a role [37]. Self-report methods are valuable, however, particularly for the assessment of activity setting and mode of activity behaviours and its determinants, which may be more difficult to assess objectively [38]. Accelerometry is the most commonly used objective method of physical activity assessment in youth. The combination of measuring movement (through accelerometry) and physiological measures (such as heart rate monitoring) may provide a more accurate estimation of physical activity energy expenditure (PAEE) than using movement sensor technology alone [39]. However, the advantages in both the accuracy and quality of information collected through objective measuring may be negated by feasibility issues of data collection such as time, participant burden and cost of equipment [38].

#### Accelerometry (Actiheart)

PA and sedentary behaviour were objectively measured using the Actiheart (Cambridge Neurotechnology Ltd, Papworth, UK). This is a lightweight and waterproof device which allows movement, as measured by accelerometry, to be recorded at the same time as heart rate. The Actiheart is reported to be technically reliable, and heart rate-physical activity intensity (HR-PAI) relationships valid against indirect calorimetry [40]. The ActiHeart is attached to the chest using two standard ECG electrodes with one electrode positioned at the base of the wearer's sternum, and the other horizontally on the wearer's left hand side. A variety of long-term paediatric electrodes were piloted. The ActiHearts were set-up to measure daily energy expenditure over a 7-day period in 60-second epochs. Children were provided with an information sheet and given oral instructions and demonstrations on how to attach the ActiHeart and electrodes correctly. A comprehensive information and instruction sheet was also provided for the parents of each child and sufficient replacement electrodes were supplied. Each child was asked to complete a diary reporting daily usage and any issues associated with non-usage of the monitor. Accelerometry-assessed PA (activity counts per minute) and heart rate data will be calculated for each child for up to a maximum of seven days.

#### Self-report recall (Youth Physical Activity Questionnaire)

Subjective measures of frequency, duration, intensity and mode of PA and frequency of sedentary behaviour, in both school and leisure time, were made with an adapted version of the Youth Physical Activity Questionnaire (YPAQ) [38]. Piloting of the original YPAQ suggested children had difficulty with the cognitive recall ability and the numerical skills required to obtain total hours and minutes values for each activity. Consequently, an adapted version, developed for the SPEEDY trial [41] was used in the main study. Participants were asked to recall frequency (never, once, 2-3 times and 4 or more times) and duration of each activity performed, for 47 activities over the previous seven days. The questionnaire was completed in the presence of a researcher in order to ascertain the level of assistance required for comprehension, and length of time necessary for completion. Data will be used to calculate time spent in moderate-to-vigorous physical activity (MVPA) and to estimate PAEE.

#### Dietary intake

##### 24 h recalls

Food and drink consumption was estimated using multiple pass 24 hour recall [42]. Multiple pass refers to the number of times (usually at least three) that the interviewer goes through the information, obtaining by means of each pass - 1) a quick list of foods consumed, then 2) a detailed description of each food and amount and 3) a review to ensure nothing has been omitted. Each child completed one recall and the acceptability of a second recall was explored among a sub-set of participants.

##### Food diaries

Participants also completed a 3-day diet diary to test the feasibility of a more detailed measure of diet and, potentially, to provide calibration of the 24 h recalls. The diaries were simple and free form to include all food and drink consumed for 3 days (including one weekend day) and the time of each eating occasion.

##### Portion size estimation

Detailed information on ethnic differences in children's food portion sizes is sparse and therefore the appropriateness of 'average' portion size data for multi-ethnic UK samples is unknown. A novel interactive portion size assessment system (IPSAS) - a computer-based system depicting photographs of food used and food left over to indicate portion size - has been developed by the Human Nutrition Research Centre, Newcastle University with the Food Standards Agency (FSA). In collaboration with Newcastle University, IPSAS was expanded with traditional foods of the ethnic groups in DEAL and portion size data collected at interview. The portion size selected is automatically recorded and stored in a format which can be exported into a database or statistical software [43]. Low percentage error in nutrient content of food of known weight with portion size assessed using IPSAS has been reported among children as young as 4-6 years [43].

Digital cameras offer the potential to assist in portion size estimates [44]. Children were given digital cameras (Vivitar Vivicam 3188) and instructed to take photographs of their food and drinks, prior to and following consumption, including pictures of any leftovers. Foldable 30 cm rulers were provided for inclusion in the pictures to indicate scale. A demonstration of the correct procedure was given during a supervised practice session. Written instructions to accompany the cameras were also provided.

##### Dietary analysis

Food intake data will be coded and daily intake of foods and nutrients calculated using a computerised suite of programs based on the 'Composition of Foods' food tables and supplements. Analyses will be repeated including and excluding those with implausible reports of dietary energy intake calculated on an individual basis. The aim will be to assess the quality of the data in relation to completeness from the two different measures of food and nutrient consumption.

##### Self efficacy (SE)

Increasing self efficacy is seen as a key facilitator to implementing behaviour change [45]. We explored the development of educative/motivational strategies (e.g. appropriate goal setting) to address psycho-social issues.

##### PA SE

Two measures of self efficacy for PA were piloted. A modified version of the eight item questionnaire designed by Motl and colleagues [46] was used to assess perceptions of confidence in the ability to do physical activity. The questionnaire is rated on a 5-point Likert scale ranging from 1 'I'm sure I can't' to 5 'I'm sure I can'. In addition a 22-item questionnaire was piloted using an identical 5-point scale. This questionnaire was adapted from Saunders and colleagues[47] and also includes four, single item, behaviour specific questions based on work by Timperio and colleagues [48].

##### Dietary SE

The dietary self-efficacy measure was based on a questionnaire from the Promoting Activity and Changes in Eating (PACE) study [49] and work by the Newcastle Human Nutrition Research Centre [50]. Participants were asked to rate the extent to which they were confident they could limit fatty foods, and select and request fruit, vegetables and fruit juice, including as substitutes to less healthy snacks and drinks. The 10-item questionnaire is rated on a 5-point scale consistent with the PA self-efficacy questionnaire (i.e. 1 'I'm sure I can't' to 5 'I'm sure I can').

## Discussion

### Emerging themes

Analysis of the data is ongoing, nonetheless broad themes are emerging. It is apparent that the school setting may be better for the implementation of a healthy lifestyle intervention. This is due to the ready access to large numbers from the target ethnic groups, the potential for linking interventions with the school curriculum but also due to researchers being known to schools and having experience of working within these environments. The contrast between dialogue in schools and in the places of worship, however, serves to highlight the complexities of cultural identities that the study participants negotiate on a daily basis. There appears to be freer discussion of traditional food practices among some in the places of worship compared to the school, while others were keen to stress their interest in and enjoyment of traditional foods at home in addition to less traditional foods and a desire to have foods from a wide range of cultures at school. Places of worship provide access to the wider family and therefore offer valuable opportunities for family and culturally specific support for the implementation of the intervention.

This study will continue to have active involvement of the main stakeholders, parents and children, as their perspectives will be used to develop the interventions that they think are acceptable and sustainable. In DASH, several civic leaders were helpful in recruiting the schools and respondents and we built on this goodwill to help with recruitment and awareness raising. It became evident that an intervention implemented via places of worship cannot be thought of in isolation of additional supportive community mechanisms, local authority funded (e.g. community diversity officers, inter-faith forums) and charity funded networks. This is particularly important for temples and mosques where the administrative organisation is supported by the Charities Act and is likely to be a key factor in supporting the implementation of the planned intervention.

Recruitment of the grandparents presented the additional challenge in ensuring informed consent. Materials were translated into the required languages, in addition to providing them in English, however, many older ethnic minority groups are unable to read and write, including in their own language. There may be further barriers beyond language, for example concepts of health and its determinants may differ from the dominant medical model [51]. Written contracts may be mistrusted or not endorsed in cultures where verbal communication is heavily relied on and communal patterns of decision making may be more common. Consent in these circumstances may be a continuing process of negotiation between researcher and informant which requires a relationship of trust [51].

### Public Health links

We will build on our links with the Food Standards Agency, Health Promoting Schools network and School Physical Activity co-ordinators. The Public Health Research Consortium (PHRC), through which the study is funded by the Department of Health, has strong links with the Young People's Reference Group on Public Health and INVOLVE (a national advisory group funded by the National Institute for Health Research to support and promote active public involvement in NHS, public health and social care research). These links will be further explored during the remainder of the study and in future work.

### Policy relevance

Halting the rise in childhood and adolescent obesity is a policy priority as reflected in a range of government initiatives (e.g. Choosing Health; Choosing Activity; Choosing a better Diet: A Food and Action Plan; Healthy Schools Programme; Every Child Matters: Change for Children Programme) with specific actions on diet and physical activity in schools and in the community. Little, however, is known about what promotes or hinders engagement with prevention programmes among ethnic minority children. This study will enhance policy initiatives by developing the evidence base about culturally acceptable interventions to reduce the risk of obesity in childhood and adolescence.

## Abbreviations

**BHF**: British Heart Foundation; **DASH**: Determinants of adolescent Social wellbeing and Health study; **DEAL**: DiEt and Active Living study; **FSA**: Food Standards Agency; **INVOLVE**: A national advisory group funded by National Institute for Health Research; **IPSAS**: Interactive portion size assessment system; **MVPA**: Moderate-to-Vigorous Physical Activity; **PA**: Physical activity; **PACE**: Promoting Activity and Changing Eating; **PAEE**: Physical Activity Energy Expenditure; **PHRC**: Public Health Research Consortium; **POW**: Place of worship; **YPAQ**: Youth Physical Activity Questionnaire.

## Competing interests

The authors declare that they have no competing interests.

## Authors' contributions

SH is the PI and leads the study. SH and MM conceived the study and MM prepared the first draft of the manuscript. All authors participated in subsequent drafts of the manuscript and approved the final version. GB and ER conducted the fieldwork.

## Pre-publication history

The pre-publication history for this paper can be accessed here:

http://www.biomedcentral.com/1471-2458/9/480/prepub
